# Cardioprotective effects of miR-34a silencing in a rat model of doxorubicin toxicity

**DOI:** 10.1038/s41598-020-69038-3

**Published:** 2020-07-23

**Authors:** Elena Piegari, Anna Cozzolino, Loreta Pia Ciuffreda, Donato Cappetta, Antonella De Angelis, Konrad Urbanek, Francesco Rossi, Liberato Berrino

**Affiliations:** 1 Department of Experimental Medicine, Section of Pharmacology, University of Campania “Luigi Vanvitelli”, Via Costantinopoli 16, 80138 Naples, Italy; 2 Department of Experimental and Clinical Medicine, University “Magna Graecia” of Catanzaro, Viale Europa, 88100 Catanzaro, Italy

**Keywords:** Pharmacology, miRNAs, Cardiovascular diseases

## Abstract

Cardiotoxicity remains a serious problem in anthracycline-treated oncologic patients. Therapeutic modulation of microRNA expression is emerging as a cardioprotective approach in several cardiovascular pathologies. MiR-34a increased in animals and patients exposed to anthracyclines and is involved in cardiac repair. In our previous study, we demonstrated beneficial effects of miR-34a silencing in rat cardiac cells exposed to doxorubicin (DOXO). The aim of the present work is to evaluate the potential cardioprotective properties of a specific antimiR-34a (Ant34a) in an experimental model of DOXO-induced cardiotoxicity. Results indicate that in our model systemic administration of Ant34a completely silences miR-34a myocardial expression and importantly attenuates DOXO-induced cardiac dysfunction. Ant34a systemic delivery in DOXO-treated rats triggers an upregulation of prosurvival miR-34a targets Bcl-2 and SIRT1 that mediate a reduction of DOXO-induced cardiac damage represented by myocardial apoptosis, senescence, fibrosis and inflammation. These findings suggest that miR-34a therapeutic inhibition may have clinical relevance to attenuate DOXO-induced toxicity in the heart of oncologic patients.

## Introduction

The anthracycline doxorubicin (DOXO) is a very powerful antineoplastic drug whose clinical use is limited by cardiotoxicity, its main side effect that may occur both acutely and chronically, affecting the quality of life of otherwise successfully treated oncologic patients^1,2^. Anthracycline cardiotoxicity begins with subclinical myocardial damage, progresses to an early asymptomatic deterioration in left ventricle (LV) ejection fraction (EF) and ends, if not properly treated, with a symptomatic and often intractable heart failure (HF). For what concerns myocardial function, diastolic dysfunction may represent a precocious manifestation of DOXO cardiotoxicity, associated with ventricular relaxation and chamber wall stiffness leading to an alteration of ventricular function that first involves diastole and then eventually affects systole^3–6^. Given the growing successful of chemotherapy with the significant increase in cancer survival, the clinical significance of DOXO cardiotoxicity is by no means small^7^. Therefore, there is a serious need of an efficacious cardioprotective strategy to prevent or reduce ventricular complications.

MicroRNAs (miRNAs) are small noncoding RNAs that suppress protein expression through binding and silencing specific mRNAs. A single miRNA inhibits many different mRNAs simultaneously, thus allowing an amplification of biological responses. A fine manipulation of miRNA expression and function through systemic or local delivery of miRNA inhibitors (antimiRs) or mimics, has triggered the interest for miRNAs as innovative therapeutic targets^8,9^. MiRNAs are emerging as a novel treatment for cardiovascular diseases^9–11^ and recently, several studies have investigated the role of miRNAs in DOXO-induced cardiotoxicity^12,13^. MiR-34a is involved in several cellular processes, such as apoptosis, senescence and energy metabolism^14,15^ and is recognized as a key regulator in cardiac diseases and repair^16–20^. Current studies revealed increased miR-34a levels in tissue and plasma of different models of DOXO-induced cardiotoxicity^19,21–23^ and in plasma of oncologic patients after anthracycline chemotherapy^22,24,25^. Notably, in our previous study, we demonstrated that an antimiR complementary to miR-34a (Ant34a) was able to revert cardiotoxic effects of DOXO in vitro^19^. In particular, miR-34a silencing provoked an increase of its prosurvival targets Bcl-2 and SIRT1, which positively affected cell behaviour, thus increasing vitality, proliferation, senescence and apoptosis of DOXO-treated rat cardiac progenitor cells. Moreover, Ant34a treatment decreased negative paracrine effects of miR-34a on rat cardiomyocytes, fibroblasts and endothelial cells. The intrinsic ability of miR-34a to modulate different DOXO-related pathways in cardiac cells makes its inhibition an attractive therapeutic perspective. These findings prompted us to assess the potential cardioprotective effect of Ant34a in vivo. Therefore, in the present work, we evaluated the consequences of miR-34a silencing on DOXO-related pathways and importantly on cardiac function in a rat model of DOXO-induced cardiotoxicity.

## Results

### Ant34a ameliorated cardiac function reducing miR-34a levels in heart of DOXO-treated rats

**Figure 1:**
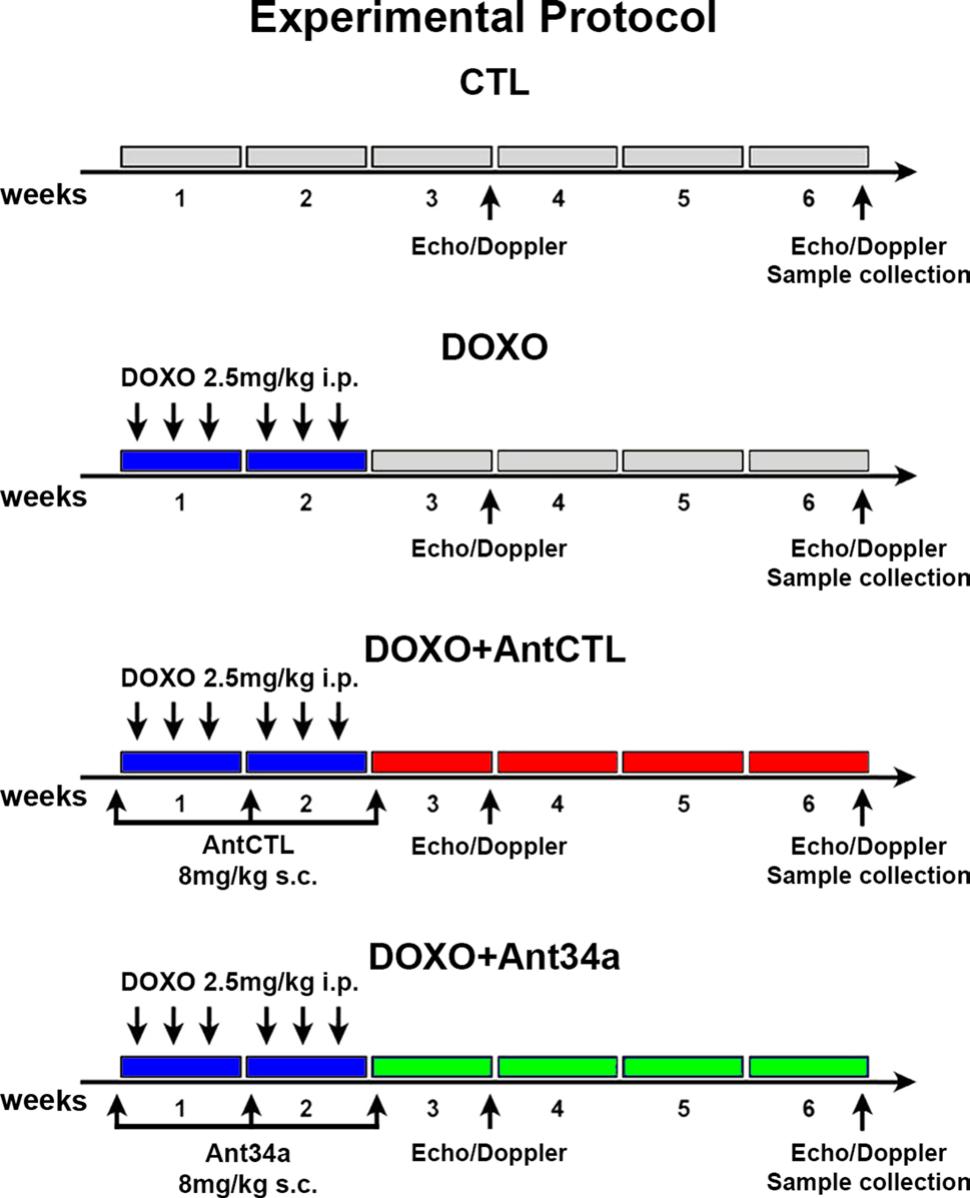
Experimental protocol. Temporal scheme of in vivo experiments.

**Figure 2:**
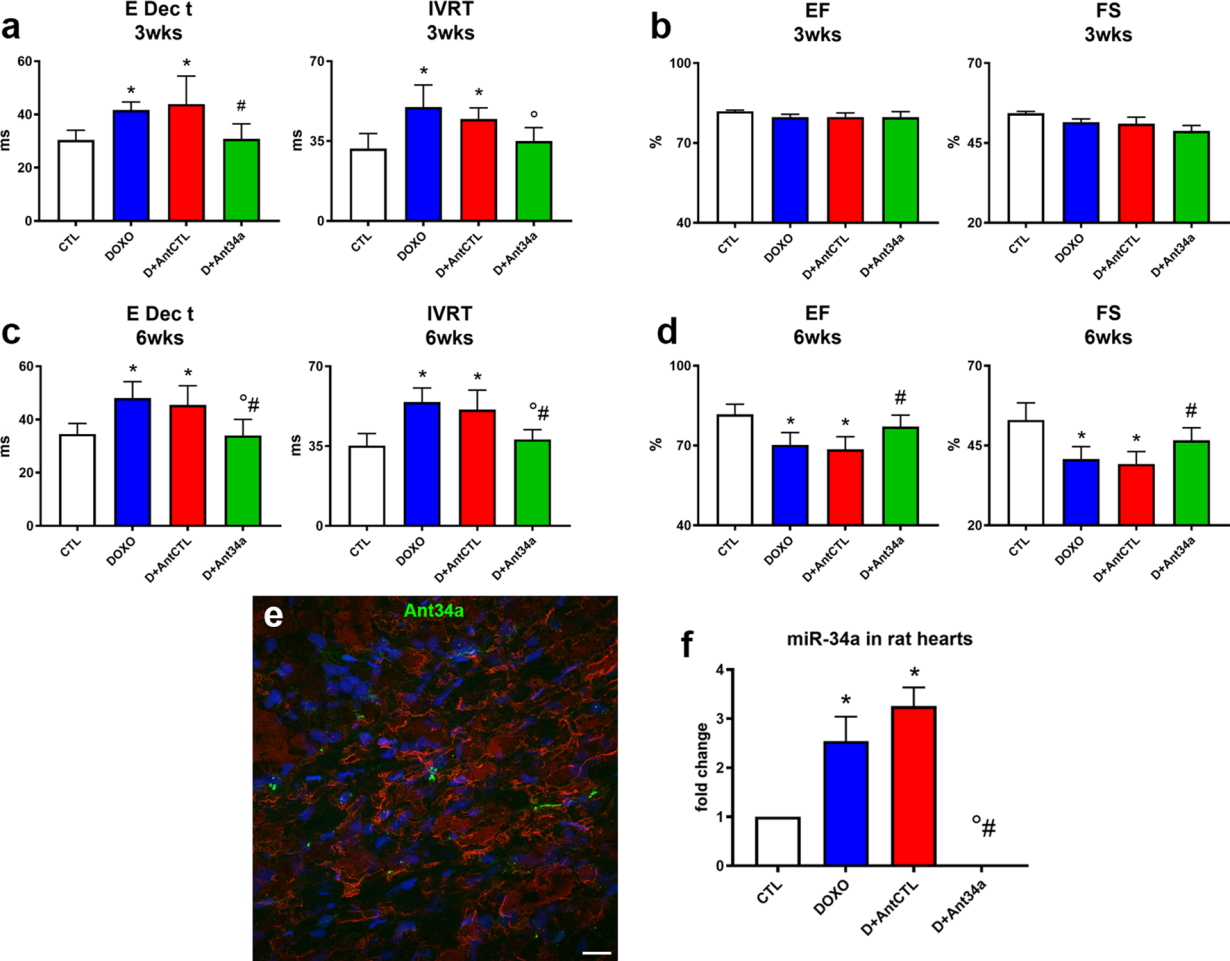
Ant34a ameliorated cardiac function reducing miR-34a levels in hearts of DOXO treated rats. (**a**) Pulsed-wave Doppler at 3 weeks showing diastolic function: peak velocity of E Dec t and IVRT. (**b**) Echocardiographic systolic indices EF and FS at 3 weeks. (**c** ) Pulsed-wave Doppler at 6 weeks showing diastolic function: peak velocity of E Dec t and IVRT. (**d**) Echocardiographic systolic indices EF and FS at 6 weeks. (**e**) The presence in the myocardium of Ant34a, labelled with the FAM fluorophore (green), is shown by histological analysis. Cardiomyocytes are labelled with α-SA (red), nuclei are stained with DAPI (blue). Scale bar 25 μm. (**f**) qPCR analysis indicated miR-34a expression in cardiac tissues of DOXO-treated rats sacrificed at 6 weeks. MiRNA expression is shown as fold change with respect to CTL group. Results are presented as mean ± SD, n = 5–10/group. *p < 0.05 vs CTL; °p < 0.05 vs DOXO; ^#^p < 0.05 vs D + AntCTL. D: DOXO.

The potential cardioprotective properties of miR-34a silencing were assessed in the well-known model of DOXO-induced cardiotoxicity^26–31^. Rats received 6 intraperitoneal injections of 2.5 mg/kg DOXO over a period of 2 weeks and Ant34a or AntCTL were administered subcutaneously by 3 injections of 8 mg/kg, 1 day before DOXO treatment, and at day 7 and 14 of DOXO schedule (Fig. 1). Cardiac function was evaluated at 3 and 6 weeks after the first injection of DOXO. As previously demonstrated, diastolic and systolic functions progressively deteriorated in DOXO-treated animals^27,31^. In fact, echocardiographic measurements indicated that, at 3 weeks from the first injection of DOXO, an early worsening of cardiac function appeared in DOXO and DOXO + AntCTL groups. In particular, diastolic function was significantly deteriorated as evidenced by the increase of E Dec T and IVRT parameters (Fig. 2a), measured following pulsed-wave Doppler, while systolic one evaluated by EF and FS did not show significant variations (Fig. 2b). At 6 weeks after the first injection of DOXO, in DOXO and DOXO + AntCTL groups, diastolic function progressively worsened (Fig. 2c) and also systolic function significantly changed, as evidenced by the reduction in EF and FS (Fig. 2d). Importantly, Ant34a systemic administration in DOXO-treated animals significantly improved cardiac function, ameliorating diastolic function already at 3 weeks, as evidenced by the reduction of E Dec T and IVRT parameters (Fig. 2a), and also improving systolic function at 6 weeks as shown by the values of EF and FS (Fig. 2d). To verify that the beneficial effects on DOXO-induced cardiac dysfunction were mediated by miR-34a silencing, Ant34a cardiac uptake and efficacy in inhibiting its target miRNA were evaluated. Histological analysis confirmed the presence in the myocardium of FAM-labelled Ant34a, indicating that the antimiR was taken up by cardiac tissue (Fig. 2e). Consistent with these results, RT-PCR analysis showed that although miR-34a levels raised up by 3.26 fold in hearts of DOXO-treated animals sacrificed at 6 weeks after the first injection of DOXO, Ant34a administration completely silenced miR-34a cardiac expression in DOXO + Ant34a group (Fig. 2f).

### Ant34a upregulates cardiac expression of Bcl-2 and SIRT1 in DOXO-treated rats

**Figure 3:**
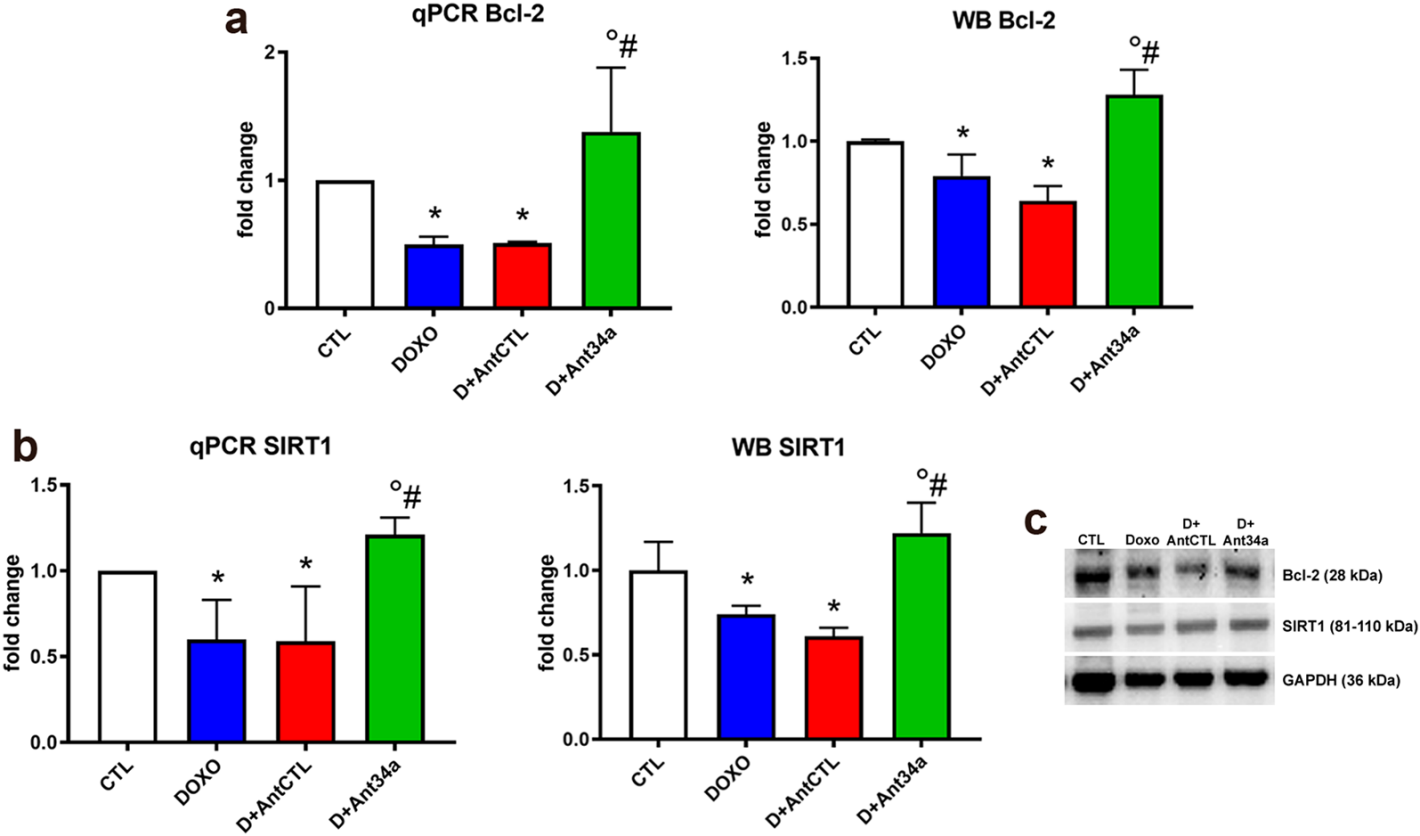
Ant34a upregulates cardiac expression of Bcl-2 and SIRT1 in DOXO treated rats. (**a**) Bcl-2 mRNA and protein levels were evaluated by qPCR and western blot analysis respectively. (**b**) SIRT1 mRNA and protein levels were evaluated by qPCR and western blot analysis respectively. (**c**) Representative images of western blot protein bands. Full-length blots are displayed in Supplementary Fig. S1a–b. mRNA and protein expressions are shown as fold change with respect to CTL group. Results are presented as mean ± SD, n = 5–8/group. *p < 0.05 vs CTL; °p < 0.05 vs DOXO; ^#^p < 0.05 vs D + AntCTL. D: DOXO.

To examine the mechanisms by which Ant34a could mediate positive effects on DOXO-induced myocardial dysfunction, cardiac expression of Bcl-2 and SIRT1, among miR-34a targets and both involved in DOXO-related pathways, was evaluated. Although DOXO prompted a significant decrease in cardiac Bcl-2 and SIRT1 mRNA and protein levels in DOXO and DOXO + AntCTL groups, Ant34a administration significantly increased both Bcl-2 and SIRT1 myocardial expression in DOXO + Ant34a animals as validated by qPCR and western blot analysis respectively (Fig. 3a–c). Therefore, to assess if Bcl-2 and SIRT1 raises mediated by miR-34a silencing could positively interfere with DOXO-induced cardiotoxicity, their DOXO-related downstream pathways were analysed.

### Ant34a reduces myocardial apoptosis and senescence in DOXO-treated rats

**Figure 4:**
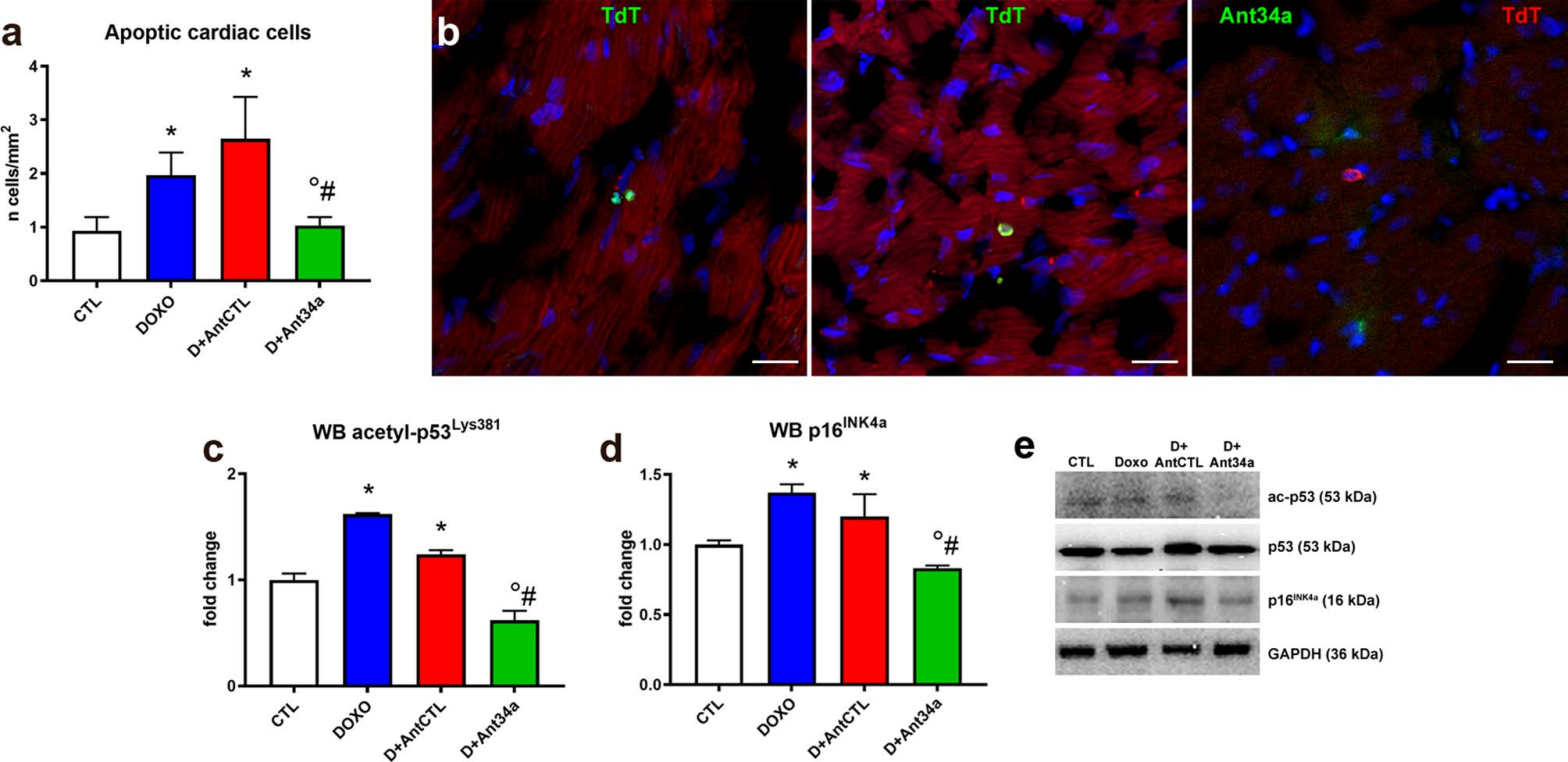
Ant34a regulates myocardial apoptosis and senescence in DOXO treated rats. (**a**) Cardiac apoptosis measured by TdT assay. (**b**) Cardiac apoptic cells within the myocardium of DOXO (left panel, TdT green) and Ant34a treated (central panel, TdT green; right panel, TdT red) animals. Cardiomyocytes are labelled with α-SA (red), nuclei are stained with DAPI (blue). Scale bars 20 μm. (**c**) Levels of acetyl-p53^Lys381^ were evaluated by western blot analysis. (**d**) p16^INK4a^ expression was examined by western blot analysis. (**e**) Representative images of western blot protein bands. Full-length blots are displayed in Supplementary Fig. S1d–g. Protein expressions are shown as fold change with respect to CTL group. Results are presented as mean ± SD, n = 5–8/group. *p < 0.05 vs CTL; °p < 0.05 vs DOXO; ^#^p < 0.05 vs D + AntCTL. D: DOXO.

Because Ant34a administration in DOXO-treated animals triggered an upregulation of the antiapoptotic protein Bcl-2, miR-34a silencing effect on cardiac DOXO-induced apoptosis was evaluated by TdT assay. In comparison with DOXO animals, Ant34a treatment resulted in a lower number of cardiac cells undergoing apoptosis (Fig. 4a, b). Apoptotic event are also modulated by activation of p53, one of the non-histonic targets deacetylated by SIRT1. A positive feedback loop regulates p53-miR-34a-SIRT1 axis: p53 induces miR-34a and miR-34a activates p53 by inhibiting SIRT1 expression^14,32^. Lower levels of acetylp53^Lys381^ in myocardial tissue of DOXO + Ant34a group suggest the involvement also of SIRT1 in turning off pro-apoptotic signalling activated by DOXO (Fig. 4c, e).

Since SIRT1-mediated deacetylation of p53 may also influence the cellular senescence process^27,33^, the cardiac expression of p16^INK4a^ was examined. DOXO induced an increase of p16^INK4a^ in DOXO and DOXO + AntCTL groups, but Ant34a treatment resulted in a significant reduction in p16^INK4a^ expression (Fig. 4d, e). Thus, miR-34a inhibition could protect from DOXO-induced cardiac apoptosis and senescence.

### Ant34a moderates myocardial fibrosis and inflammation in DOXO-treated rats

**Figure 5:**
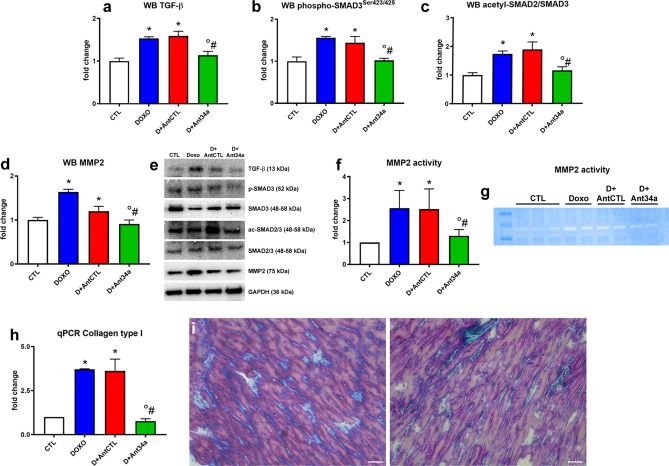
Ant34a reduces myocardial fibrosis in DOXO treated rats. (**a**–**d**) Quantification of TGF-β, phospho-SMAD3^Ser423/425^, acetyl-SMAD2/SMAD3 and MMP2 protein expression levels. (**e**) Representative images of western blot protein bands. Full-length blots are displayed in Supplementary Fig. S1h–n. (**f**) Quantification of MMP2 gelatinolytic activity. (**g**) Representative image of MMP2 zymography. (**h**) Collagen type 1 mRNA levels analysed by qPCR. (**i**) Masson's trichrome staining showing collagen deposition (blue) in the hearts of DOXO exposed (left panel) and cale bars 100 μm. mRNA and protein expressions and MMP2 activity are shown as fold change with respect to CTL group. Results are presented as mean ± SD, n = 5–8/group. *p < 0.05 vs CTL; °p < 0.05 vs DOXO; ^#^p < 0.05 vs D + AntCTL. D: DOXO.

ROS production induced by DOXO trigger TGF-β pathway that activates cardiac fibroblasts promoting fibrosis^30,34^. The effects of miR-34a silencing on pathways that underlie DOXO-induced cardiac fibrosis were examined. DOXO and DOXO + AntCTL animals exhibited significant increases in pro-fibrotic markers TGF-β and its downstream effector phospho-SMAD3 (Fig. 5a, b, e), but Ant34a treatment significantly reduced the levels of TGF-β and diminished phospho-SMAD3^Ser423/425^/SMAD3 ratio (Fig. 5a, b, e). Since SMAD2/SMAD3 represent a molecular target of deacetylase SIRT1^35^, the acetylated form of this complex was evaluated. Results showed a significant reduction of acetyl-SMAD2/SMAD3 consequent to Ant34a administration in DOXO rats (Fig. 5c, e), indicating the involvement of SIRT1 activation, mediated by miR-34a inhibition, in the observed pathway. Moreover, the cardiac expression and activity of MMP2, the main enzyme involved in the remodeling of the extracellular matrix, were increased after DOXO treatment (Fig. 5d–g). Interestingly, Ant34a was able to revert these changes (Fig. 5d–g). Finally, qPCR and cardiac histochemical analysis confirmed the beneficial effect of miR-34a inhibition on DOXO-activation of fibrosis process. In fact, the results showed that in hearts of DOXO animals, collagen type 1 mRNA expression and collagen deposition were increased, whereas Ant34a administration determined a significant reduction (Fig. 5 h, i).

**Figure 6:**
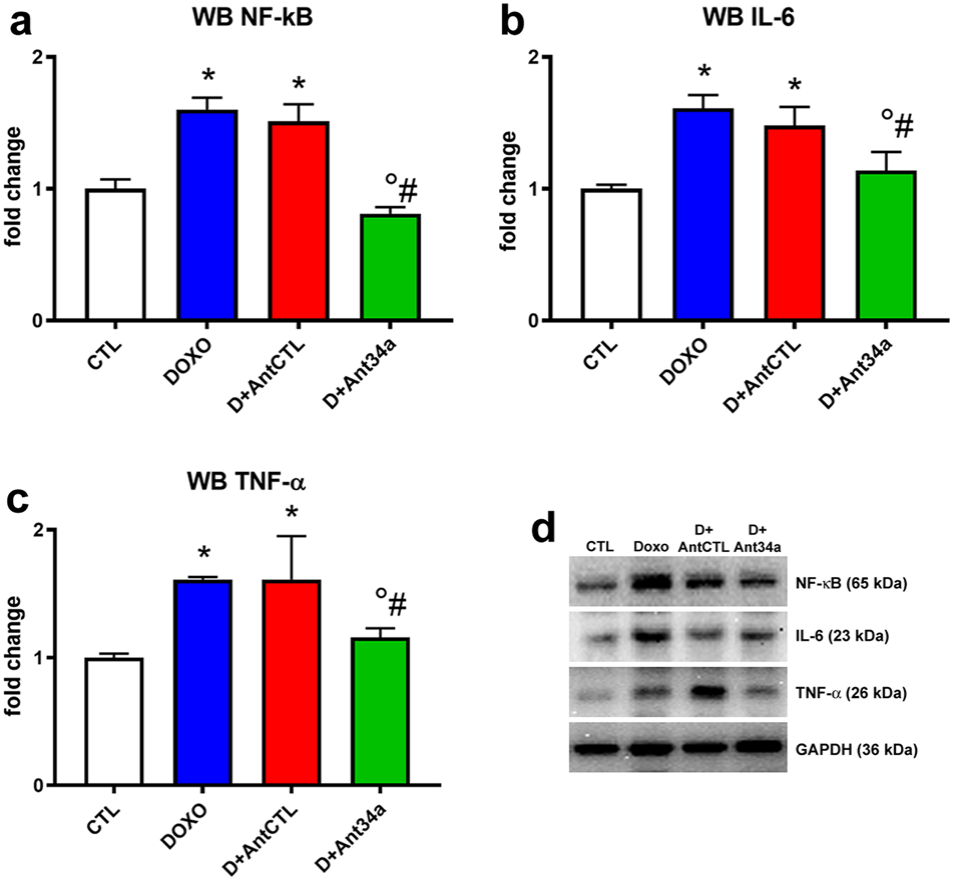
Ant34a reduces cardiac inflammation in DOXO treated rats. (**a**–**c**) Quantification of NF-κB, IL-6 and TNF-α protein expression levels. (**d**) Representative images of western blot protein bands. Full-length blots are displayed in Supplementary Fig. S1o–r. Protein expressions are shown as fold change with respect to CTL group. Results are presented as mean ± SD, n = 5–8/group. *p < 0.05 vs CTL; °p < 0.05 vs DOXO; ^#^p < 0.05 vs D + AntCTL. D: DOXO.

The extent of fibrosis is determined by a balance between pro-fibrotic and pro-inflammatory signals such as TNF-α, IL-1β, IL-6 and anti-inflammatory signals^36^. NF-κB, a major determinant of the inflammatory process, promotes the transcription of several pro-inflammatory genes, and notably induces miR-34a expression^37,38^. MiR-34a induction mediated by NF-κB lead to a SIRT1 activity suppression; in contrast, SIRT1 inhibits NF-κB activation by its deacetylation^37,39^. Therefore, to evaluate if miR-34a inhibition could positively influence the inflammatory pathway induced by DOXO, cardiac expression of NF-κB, IL-6 and TNF-α was examined. As expected, DOXO and DOXO + AntCTL groups showed an increased myocardial expression of NF-κB coupled with a higher content of its related mediators IL-6 and TNF-α (Fig. 6a–d). Ant34a administration significantly reduced NF-κB, IL-6 and TNF-α levels in hearts of DOXO animals (Fig. 6a–d), indicating its potential anti-inflammatory effect in cardiac DOXO-induced inflammation.

### Ant34a reduced miR-34a levels in liver, kidney and spleen of DOXO-treated rats

**Figure 7:**
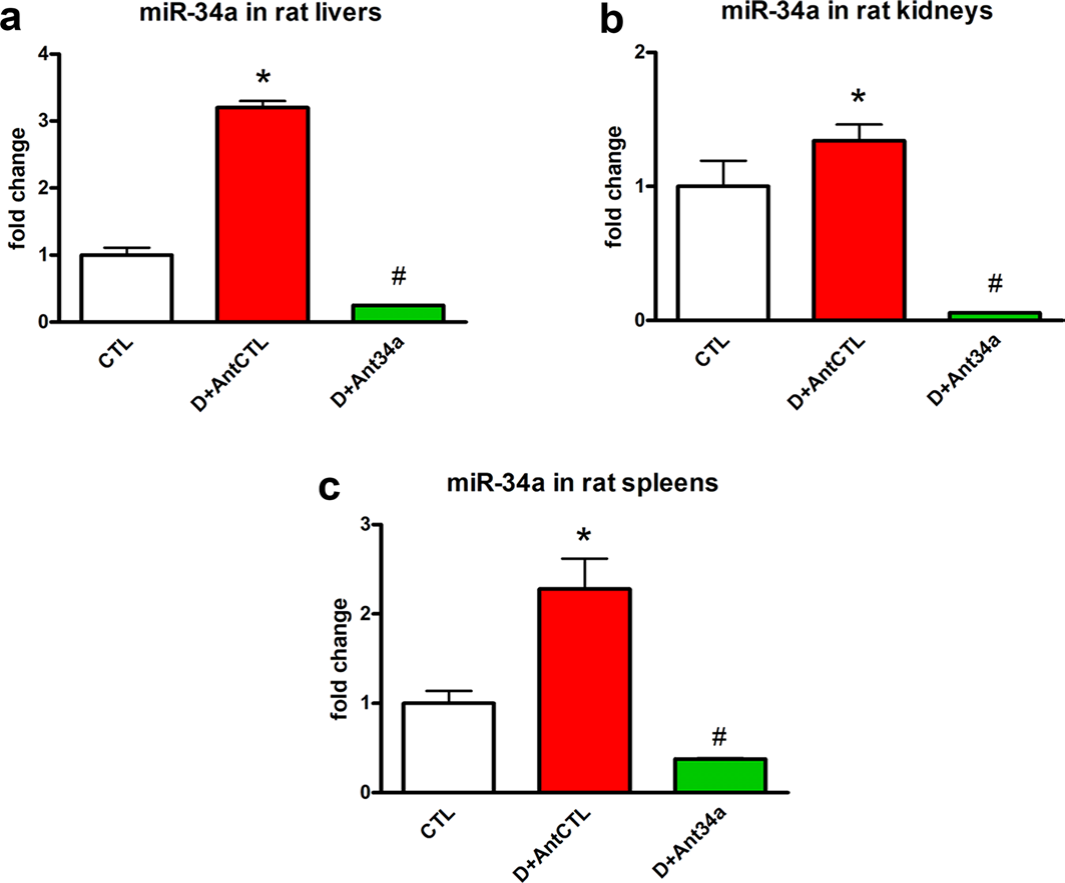
Ant34a reduced miR-34a levels in liver, kidney and spleen of DOXO-treated rats. (**a**–**c**) qPCR analysis indicated miR-34a expression in liver, kidney and spleen of DOXO-treated rats sacrificed at 6 weeks. MiRNA expression is shown as fold change with respect to CTL group. Results are presented as mean ± SD, n = 5/group. *p < 0.05 vs CTL; ^#^p < 0.05 vs D + AntCTL. D: DOXO.

To evaluate if systemic administration of Ant34a inhibited miR-34a expression also in other organs, the levels of this miRNA were analysed in liver, kidney and spleen of DOXO-treated rats. Results indicated that although DOXO administration upregulated miR-34a in such organs, Ant34a treatment drastically reduced its levels (Fig. 7a–c). Since miR-34a increase has been correlate with different organ damage activating oxidative stress and inflammatory pathways and importantly its inhibition alleviates tissue injury^40–42^, it could be taken into consideration as a novel candidate target in inflammatory and oxidative stress driven organ diseases such as DOXO-induced toxicity.

## Discussion

DOXO is a highly effective antineoplastic drug, but clinical application is hampered by cardiotoxicity, its most harmful and worrying side effect. Therefore, a search for therapeutic intervention that can early interfere with the progression of cardiotoxicity and possibly prevent the consequent development of HF is needed.

The discovery of therapeutic miRNAs, a class of small non-coding regulatory RNAs, is considered one of the most exciting and significant pharmacological innovations in drug development^9^. MiR-34a is implicated in several cellular process such as cell cycle, metabolism, proliferation and differentiation and represents a potential therapeutic target in cardiovascular diseases^15^. The cardioprotective properties of miR-34a silencing have been demonstrated in different models of cardiac pathologies^16–18,43,44^. Moreover, we have previously demonstrated that knockdown of miR-34a in cardiac progenitor cells exhibited therapeutic potential of DOXO-induced toxicity in vitro^19^. Consequently, in the current study, we explored the effects of systemic inhibition of miR-34a, by a LNA-modified antimiR, in a model of DOXO-induced cardiotoxicity.

The first important finding of this paper is the demonstration that Ant34a treatment of animals exposed to DOXO translated into significant and early cardiac functional improvement. DOXO cardiotoxicity is probably a progressive mechanism starting with cardiac injury, reduction in myocardial deformation, followed by progressive LV decline, which, if not properly treated, progressively leads to symptomatic HF. Notably, asymptomatic diastolic dysfunction can be the initial manifestation of DOXO cardiotoxicity that subsequently involves systole^3–6^. Consistently, in our experimental protocol, at the end of DOXO treatment, systolic parameters such as EF and FS did not change while altered diastolic indices defined an impaired ventricular relaxation. Despite to a progressive deterioration of diastolic and systolic functions observed in DOXO-treated rats, inhibition of miR-34a led to an amelioration of cardiac function with time, improving both diastolic and systolic parameters. In addition to improving DOXO cardiac dysfunction, Ant34a administration importantly may avoid the HF from becoming intractable preserving diastolic dysfunction that is considered as a precocious sign of cardiotoxicity.

**Figure 8:**
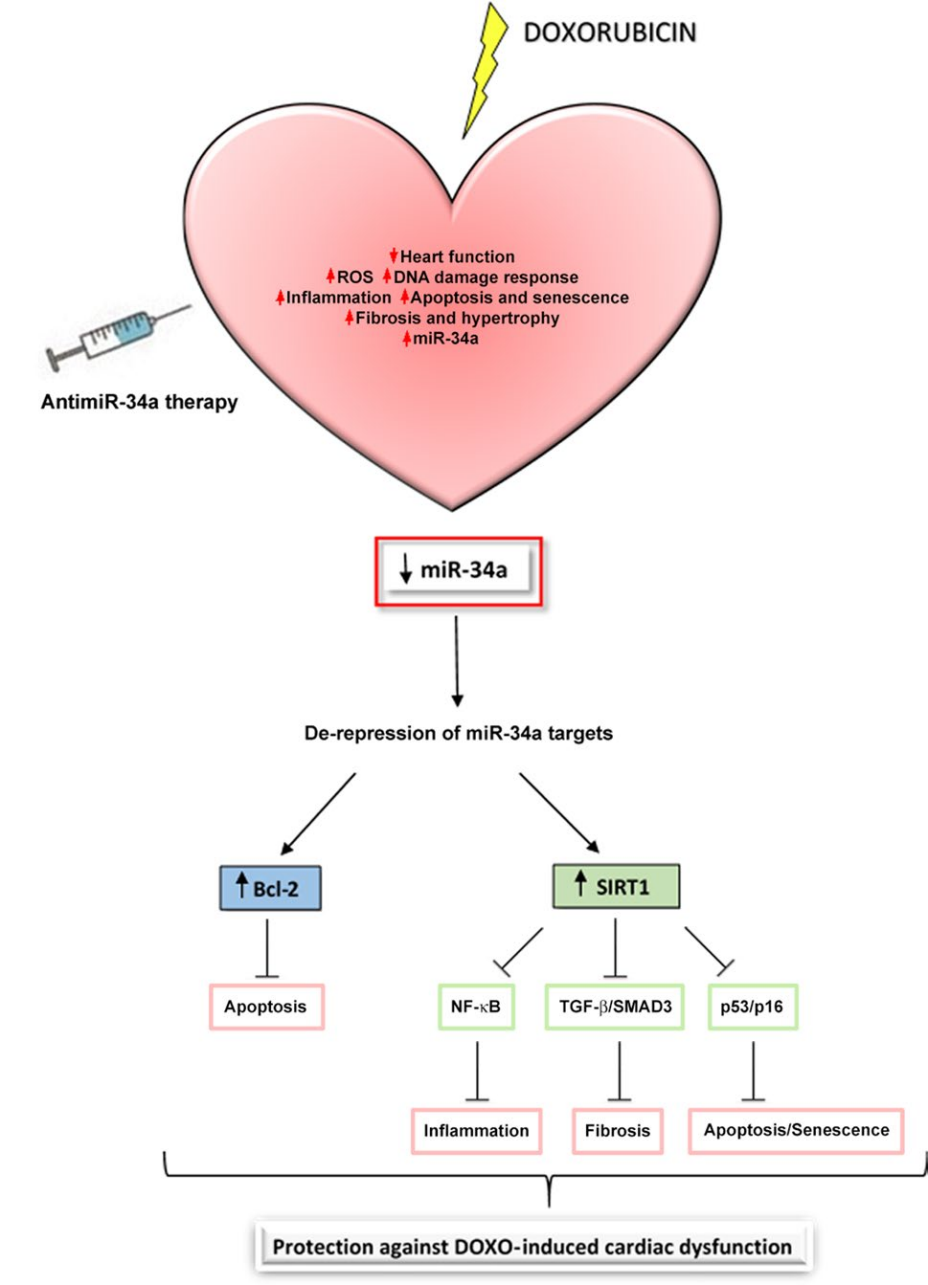
Cardioprotective mechanisms of antimiR-34a therapy. Schematic representation of the potential mechanisms by which miR-34a silencing mediate beneficial effects on cardiac dysfunction in a model of DOXO toxicity.

To deepen the molecular mechanisms that underlie Ant34a beneficial effects in DOXO cardiac dysfunction, the expression of downstream targets modulated by miR-34a, as Bcl-2 and SIRT1, was assessed. Bcl-2 is an antiapoptotic protein that promotes cellular survival also in the heart^45^ and has a role in DOXO-induced myocardial apoptosis^46,47^. SIRT1 regulates cell death/survival-related signalling, regulating the establishment and progression of HF^48^. It has been demonstrated that different compounds modulating SIRT1 have cardioprotective effects also in DOXO-induced cardiotoxicity^28,30,49^. Importantly, our recent study demonstrated that miR-34a silencing upregulates Bcl-2 and SIRT1 expression in cardiac progenitor cells after DOXO exposure in vitro^19^. Results of the present paper demonstrated that Ant34 administration in DOXO-treated rats, by de-repressing miR-34a targets in the heart, modulated their downstream pathways (Fig. 8). In particular, Bcl-2 activation led to a significant reduction of DOXO-induced cardiac apoptosis while stimulation of SIRT1, decreasing the acetylation levels of its target proteins, p53, SMAD2/3 and NF-κB, inactivated their downstream pathways reducing myocardial senescence, fibrosis and inflammation generated by DOXO. Collectively, the regulation of these mechanisms could explain the positive effects of miR-34a therapeutic silencing on DOXO-induced cardiac dysfunction though is not established a succession for these events which also continuously correlate with each other. Noteworthy, p53 and NF-κB, deacetylated by SIRT1, are strong inducers of miR-34a that in turn inhibits SIRT1 expression forming positive feedback loops^15,39^. Moreover, the presence of p53 is essential for NF-κB-mediated induction of miR-34a. Therefore, miR-34a is finely controlled and connected with several factors in order to control cellular homeostasis and coordinate the balance between different cellular functions such as cell cycle, metabolism, proliferation, differentiation, inflammation and epigenetics. These cellular events administer the crucial biology of various chronic diseases including cardiovascular diseases, aging, diabetes and cancer. With the perspective of therapeutic miR-34a inhibition, the existence of several feedback regulatory mechanisms of miR-34a exhorts to consider the pathways that may control either downstream or upstream miR-34a targets or miR-34a regulators, respectively.

Although the advantage to regulate several cellular pathways simultaneously makes miRNA-based therapy suitable for the treatment of multi-pathway diseases such as DOXO cardiotoxicity, the lack of therapeutic specificity may appear as a limitation to understand the pharmacological mechanism of action. Nevertheless, from a translational perspective it could produce considerable benefits. Current HF therapies affect many downstream LV signalling targets, and drugs inhibiting the renin-angiotensin system are a typical example^50^. Therefore, given the complex and multifactorial pathogenesis of DOXO-induced cardiotoxicity, it is expected that its lack of specificity could be highly beneficial in the clinical setting.

Another question about Ant34a clinical implications remain to be determined. AntimiR treatment, silencing the tumor suppressor miR-34a, could interfere with anticancer activity of DOXO in oncologic patients. In our previous work, we demonstrated that a tenfold higher concentration of Ant34a is needed to reduce DOXO cytotoxic effect in tumor cells with respect to the concentration that protects cardiac cells in vitro^19^. So theoretically, miR-34a inhibition to impact on anthracycline cardiotoxicity in cancer patients should not influence chemotherapy. Besides, the knowledge that tumor tissues display a poorest penetrability with respect to normal tissues could support the hypothesis that a low dose of antimiR could be effective only in normal tissues. Anyway, further studies in tumor bearing animals should deepen this issue.

Main challenges for miRNA-based therapy are the delivery system and the potential off-target effects. Tissue-specific delivery to achieve sustained target inhibition is the key issues, and the therapeutic applicability of systemically administered LNA-modified antimiRs has been exhaustively validated to be efficacious in inducing a potent silencing of miRNAs in the heart^17,18,43,51–55^. In the present paper, Ant34a was systemically administered by 3 subcutaneous injections of 8 mg/kg before, halfway and at the end of DOXO treatment. Results demonstrated that Ant34a catched the required target miR-34a in myocardial tissue and prompted its inhibition until 28 days after the last antimiR injection. Regarding the cardiac targeted cell type it is important to point out that although cardiomyocytes has been considered for a long time the cellular target of DOXO-induced cardiotoxicity, cardiovascular homeostasis depends not only from cardiomyocytes, that account for less than one-third of the total number of cells within the heart, but different cellular components are involved in. Moreover, the contribution of cardiac progenitor cells and other cells types such as fibroblasts and vasculature cells in DOXO-induced cardiotoxicity has been recently demonstrated^6^. Since our previous results indicated that DOXO exposure enhanced miR-34a expressed and secreted by cardiac cells (cardiomyocytes, progenitor cells, fibroblasts and endothelial cells)^19^, miR-34a silencing is necessary in different cardiac cell types not only for modulating its intracellular expression but also for reducing its release, thus abolishing pathological cell-to-cell communication.

However, as demonstrated by our data the antimiR is also taken up by tissues throughout the body reducing miR-34a levels in other organs than hearts. While our results propose a therapeutic potential for Ant34a in DOXO-induced cardiotoxicity, clearly these possible off-target responses would need to be deepen in future studies. Nonetheless, systemic miR-34a inhibition could be an advantage for a desirable Ant34a use in DOXO-exposed patients, since in addition to myocardial toxicity, DOXO induces severe inflammatory responses and serious multi-organ side effects. Evidence demonstrated that DOXO could cause devastating systemic inflammation in vivo and toxicity to multiple organ systems such as circulatory, digestive, integumentary, urinary, nervous, reproductive system, triggering activation of TNF signaling pathway, generation of massive ROS and persistent expression of various pro-inflammatory cytokines^56,57^. Importantly, recent studies proposed an important pro-inflammatory role of miR-34a in systemic and chronic inflammation^58–60^. Our results showed that cardiac expression of inflammatory cytokines TNF-α and IL-6 are reduced in DOXO-animals treated with Ant34a. Interestingly, we have previously evidenced increased miR-34a levels in different organs of DOXO-treated rats^19^ that, as demonstrated in this paper, were significantly reduced by Ant34a administration. It is likely that, as in the heart, miR-34a could drive DOXO-harmful effects similarly in other tissues. Different organ damage have been correlate with a miR-34a increase that activate oxidative stress and inflammatory pathways^40–42^. Importantly, the inhibition of this miRNA alleviates tissue injury making it a new pharmacological target in different diseases. Although the effects driven by miR-34a decrease in organs of DOXO-treated animals should be deeply examined in future studies, these findings suggest a potential beneficial role of Ant34a also in DOXO-induced global inflammation and organ damage, making systemic miR-34a silencing a very appealing strategy for a prospective application in patients exposed to DOXO.

In summary, our data demonstrate that systemic inhibition of miR-34a offers a promising therapeutic approach in DOXO-induced toxicity since mediates cardioprotective pleiotropic beneficial effects such as reduced myocardial apoptosis, senescence, fibrosis and inflammatory response, preserving cardiac function and integrity.

## Methods

### LNA oligonucleotides

The miRCURY LNA™ microRNA Inhibitor oligonucleotides are LNA™-enhanced and contain phosphorothioate backbone bonds (Exiqon). LNA-oligonucleotides were resuspended in saline before administration. The sequence for mmu-miR-34a-5p inhibitor (Ant34a) is 5′ AGCTAAGACACTGCC 3′ and for the scramble control (AntCTL) 5′ ACGTCTATACGCCCA 3′. Ant34a is 5′ FAM labelled for in vivo detection.

### In vivo studies

The present study conformed to the National Ethical Guidelines (Italian Ministry of Health; D.L.vo 26, March 4, 2014) and was approved by the Ministry of Health (protocol n.1127/2016-PR, 22/11/2016) and by the Ethical Committee of the University of Campania “Luigi Vanvitelli”. 2–3 months old female Fisher 344 rats (Envigo) were divided in four groups: Control (CTL; n = 5); DOXO (n = 8); DOXO + Ant34a (n = 10); DOXO + AntCTL (n = 10) (Fig. 1). In all DOXO groups, cardiomyopathy was induced by 6 intraperitoneal injections of 2.5 mg/kg of DOXO (Teva Pharmaceuticals Industries) over a period of 2 weeks to reach a cumulative dose of 15 mg/kg^26–31^. 1 day before DOXO treatment, and at day 7 and 14 of DOXO schedule, DOXO + Ant34a and DOXO + AntCTL rats were treated with a subcutaneous injection of 8 mg/kg Ant34a and AntCTL oligonucleotides respectively^17,52^. Control rats were injected with saline solution. At 6 weeks after the first injection of DOXO, animals were sacrificed and hearts were collected.

### Echocardiography

Echocardiography was performed as described previously^31,61^. Briefly, at 3 and 6 weeks after the first injection of DOXO rats were anaesthetised with ketamine (100 mg/kg) and medetomidine (0.25 mg/kg) and echocardiographic parameters were collected with Vevo 770 (VisualSonics) equipped with a 25 MHz linear transducer. Body temperature was maintained at ~ 37 °C with a heating pad. Diastolic function was assessed by Doppler echocardiography. Specifically, mitral blood flow velocities were evaluated from a four-chamber apical view by using pulsed-wave Doppler with the sample volume placed at the mitral leaflet tips. Functional measurements included isovolumetric relaxation time (IVRT) and deceleration time of E wave (E Dec t). From the short-axis view, M-mode tracing of the LV at the level of the papillary muscles was obtained. The measurement of internal LV diameter, during diastole and systole, served to calculate systolic indices EF and fractional shortening (FS).

### Tissue preparation

The preparation of frozen sections was performed as described previously^31^. Isolated hearts were weighted, dissected and then placed onto a tissue mould and covered with OCT cryo-embedding medium (Bio-Optica). The cryo mould containing the tissue block was placed in a metal beaker filled with isopentane already placed in liquid nitrogen. After ensuring tissue was completely frozen, the tissue block was stored at − 80 °C, ready for sectioning. Tissue sections (10 μm thick) were generated by a Leica CM3050 S cryostat (Leica Microsystems). For molecular biology analysis, heart, liver, kydney and spleen samples were frozen in liquid nitrogen and then stored at − 80 °C.

### Immunohistochemistry

Histological sections were stained with Masson's trichrome (Sigma-Aldrich) or used for immunolabeling and confocal microscopy^30^. Apoptosis was detected by TdT assay (Clontech Laboratories). Myocytes were identified by α-sarcomeric actin (A2172, Sigma-Aldrich). Nuclei were stained with DAPI (Sigma-Aldrich). Samples were analyzed with a Leica DM5000B microscope and a Zeiss LSM700 confocal microscope.

### Quantitative real-time PCR of miR-34a from rat tissues

MiR-34a levels were analysed by quantitative real-time PCR as described previously^19^. MiRNAs were isolated from rat tissues by miRNeasy Mini Kit (Qiagen) according to the manufacturer's instructions. Reverse transcription (RT) were performed by TaqMan MicroRNA Reverse Transcription kit (Applied Biosystem) and GeneAmp PCR System 9,700. qPCR were performed by using the TaqMan Universal PCR Master Mix (Applied Biosystem) using the CFX96 Real-time system (Bio-Rad Laboratories). RT and qPCR used TaqMan® MicroRNA Assays (Applied Biosystem). Levels of miR-34a were detected and were normalized by RNU6B (U6) as internal control for miRNAs expression studies. Relative expression was calculated using the comparative cycle threshold (Ct) method (2 − ΔΔCt).

### Quantitative real-time PCR

Quantitative real-time PCR was performed as described previously^19,62^. Total RNA was extracted from rat hearts by miRNeasy Mini Kit (Qiagen) according to the manufacturer's instructions. Both cDNA synthesis and PCR were performed simultaneously by using the SuperScript III Platinum SYBR Green One- Step qRT-PCR Kit (Invitrogen) using the CFX96 Real-time system (Bio-Rad Laboratories). The transcript levels of SIRT1, Bcl-2 and collagen type I were detected and the housekeeping gene encoding hypoxanthine phosphoribosyltransferase (HPRT) was used as internal control for mRNA expression studies. The sequences for the primers used in this study are: SIRT1Fwd: CCCGGTTTCCGGGCAAACATCC; SIRT1Rev: GGCGGCAATGGGGAGGAGGA; Bcl2Fwd: CCCCCACCCCACCCCCAACC; Bcl2Rev: CCGTACCCCGCTGCCGAGGA; Collagen1A1Fwd: GTGACCTTGAGGTGGACACT; Collagen1A1Rev: CTTACCGCTCTTCCAGTCAG; HPRTFwd: TTGTTGGATATGCCCTTGACT; HPRTRev: CCGCTGTCTTTTAGGCTTTG. Relative expression was calculated using the comparative cycle threshold (Ct) method (2 − ΔΔCt).

### Western blotting

Western blotting was performed as described previously^31^. Tissue samples were lysed in buffer containing 0.1% Triton X100 and a cocktail of protease inhibitors (Sigma-Aldrich). Protein concentration was measured by Bradford assay (Bio-Rad Laboratories). Protein extracts were then separated on 8–12% SDS-PAGE and transferred onto PVDF membrane (GE Healthcare Life Sciences). Membranes were probed with primary antibodies against p16^INK4^ (ab54210), SIRT1 (ab110304), acetyl-p53^Lys381^ (ab61241), TGF-β (ab66043), SMAD3 (ab28379), IL-6 (ab9324), TNF-α (ab6671), MMP2 (ab86607) (Abcam); p53 (sc126, Santa Cruz); Bcl-2 (B9804, Sigma-Aldrich); phospho-SMAD3^Ser423/425^ (9,520), SMAD2/3 (8685S, Cell Signaling Technology); NF-κB p65 (PA5-16,545), acetyl-SMAD2/3 (PA576015, ThermoFisher). Loading conditions were determined with GAPDH (G8795, Sigma-Aldrich). Images were obtained by ChemiDoc-it 500 Imaging System (Bio-Rad Laboratories) and the optical density of the bands was analyzed with Quantity One Analysis Software (Bio-Rad Laboratories).

### Zymography

Zymography was performed as described previously^30^.Frozen tissue was mechanically homogenized on ice in lysis buffer containing 1% Nonidet P40, 0.1% SDS and protease inhibitors (Sigma-Aldrich). Equal amounts of not denatured total cellular proteins were separated by electrophoresis on precast gels (10% polyacrylamide minigels containing 0.1% gelatin gel; Life Technologies). Gels were then incubated in Renaturating Buffer and Developing Buffer according to manufacturer's instructions (Life Technologies). Staining was performed with 0.5% Coomassie Blue R-250. The gelatinolytic activity of MMP2 was shown as transparent bands against a dark blue background. Images were obtained by Gel Doc EZ Imager (Bio-Rad Laboratories) and the optical density of the bands was analyzed with Image Lab Software (Bio-Rad Laboratories).

### Data analysis and statistics

Results are presented as mean ± SD. All data were analysed with a GraphPad Prism version 5.01 statistical software package (GraphPad). Significance was determined by one-way ANOVA with Bonferroni post-test. All P values are two-sided and P < 0.05 was considered to be significant. All relative units are expressed as a fold change with the CTL group normalised to 1.

### Data availability

The authors declare that all data supporting the findings of this study are available within the article and its supplementary information files or from the corresponding author (EP) upon reasonable request.

## Supplementary information

Supplementary information.
