# Supplementary material for: Cardioprotective effects of miR-34a silencing in a rat model of doxorubicin toxicity
Source: Sci Rep. 2020 Jul 23;10:12250. doi: 10.1038/s41598-020-69038-3 (PMC7378226; doi:10.1038/s41598-020-69038-3)
Supplement: Supplementary file 1 — Supplementary information. [file 41598_2020_69038_MOESM1_ESM.pdf]

Supplementary Information

Cardioprotective effects of miR-34a silencing in a rat model of doxorubicin toxicity

Elena Piegari[1*], Anna Cozzolino[1], Loreta Pia Ciuffreda[1], Donato Cappetta[1], Antonella De Angelis[1], Konrad Urbanek[1,2], Francesco Rossi[1], Liberato Berrino[1]

[1]Department of Experimental Medicine, Section of Pharmacology, University of Campania "Luigi Vanvitelli", Via Costantinopoli 16, 80138, Naples, Italy

[2]Department of Experimental and Clinical Medicine, University "Magna Graecia" of Catanzaro, Viale Europa, 88100, Catanzaro, Italy

***Corresponding author:** Elena Piegari email: elena.piegari@unicampania.it; telephone number: +39 081 5665881

Supplementary Figure 1: Full-length images of Western blots. Red boxes indicate portions used in manuscript. In some blots, we used transfer membranes cut horizontally. 1-2-3-4 refer to CTL, DOXO, D+AntCTL, D+Ant34a.

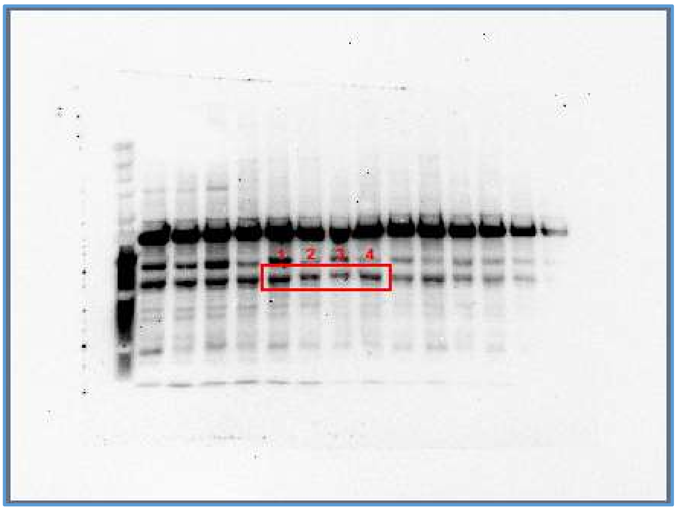

a

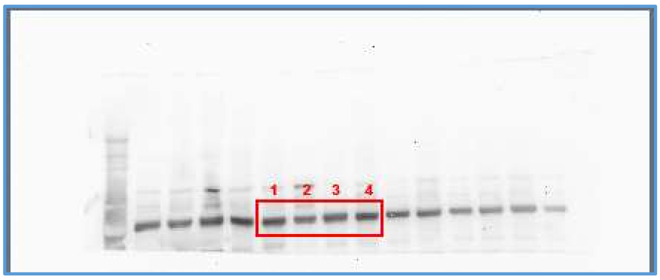

b

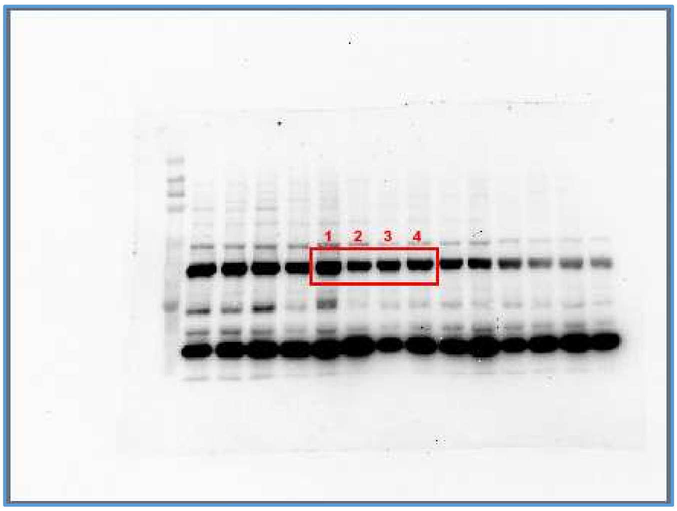

c

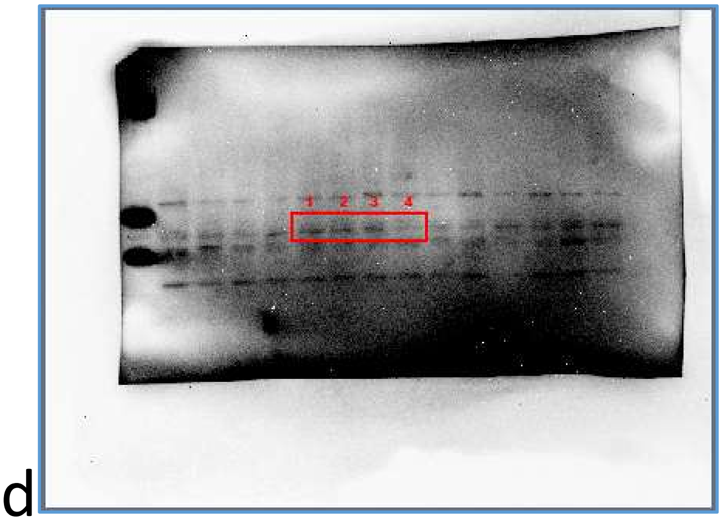

d

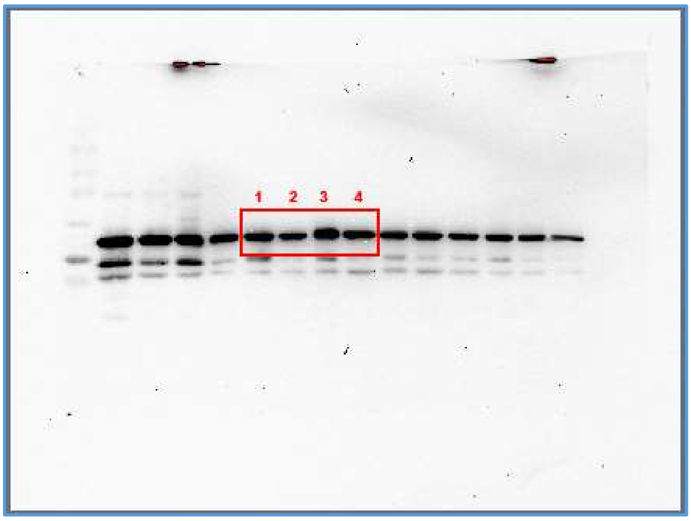

e

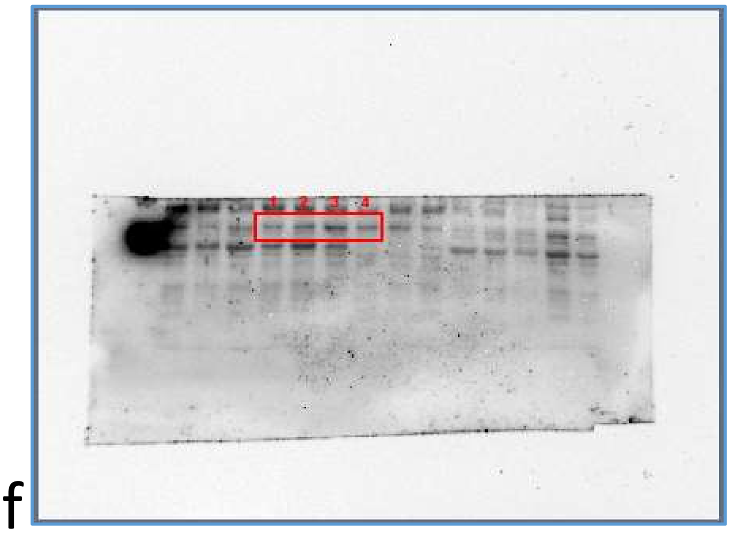

f

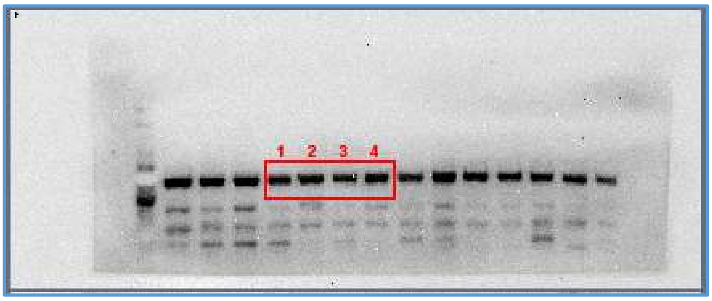

g

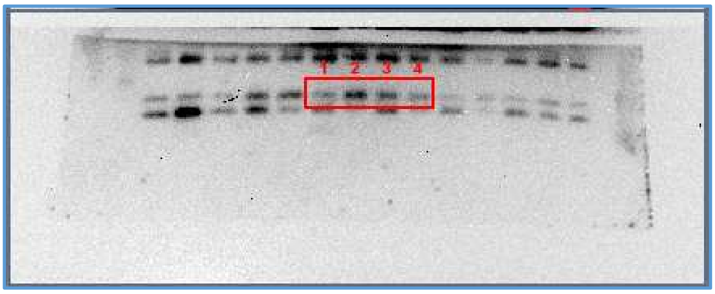

h

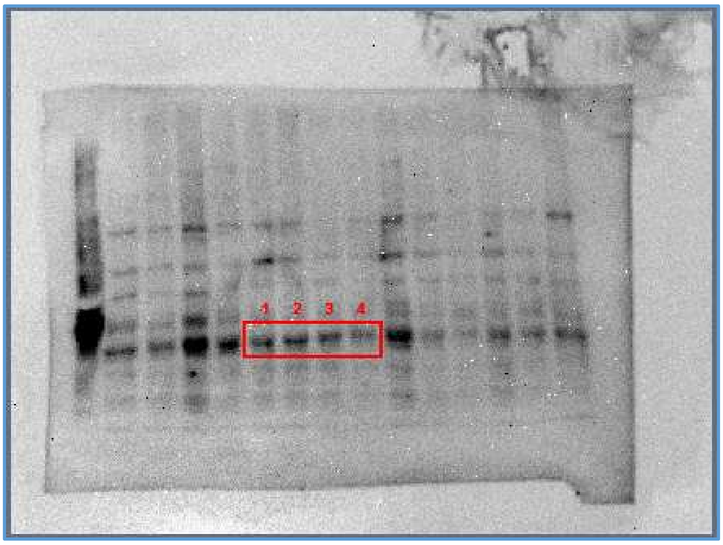

i

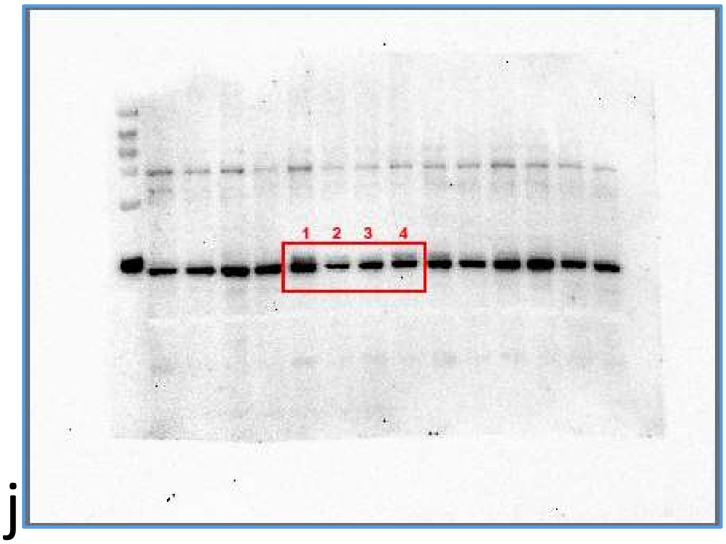

j

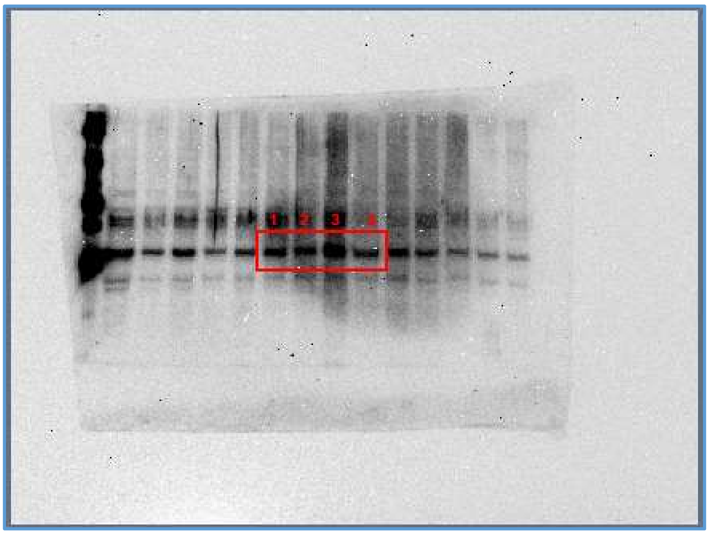

k

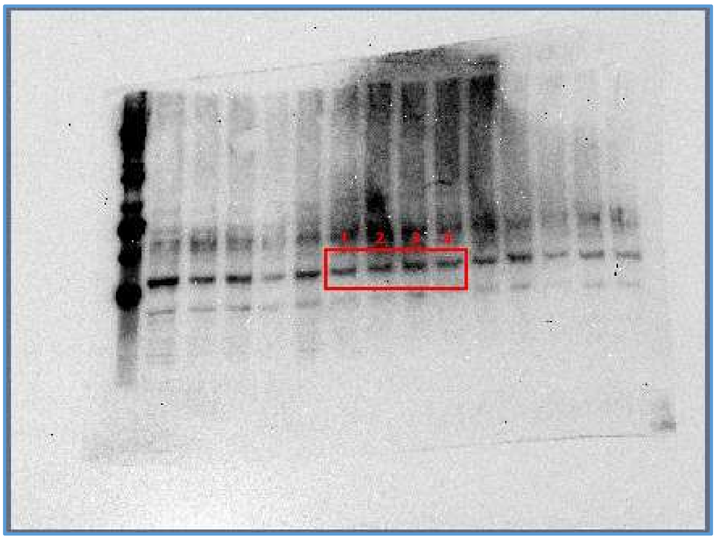

l

m

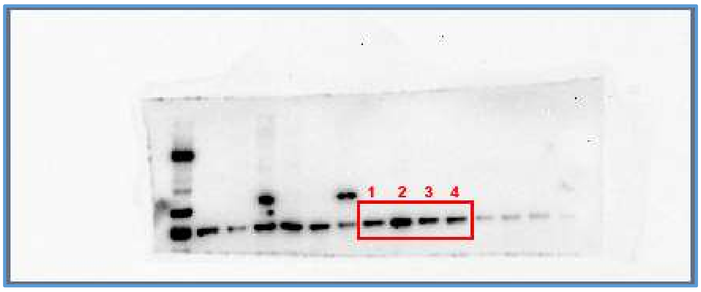

n

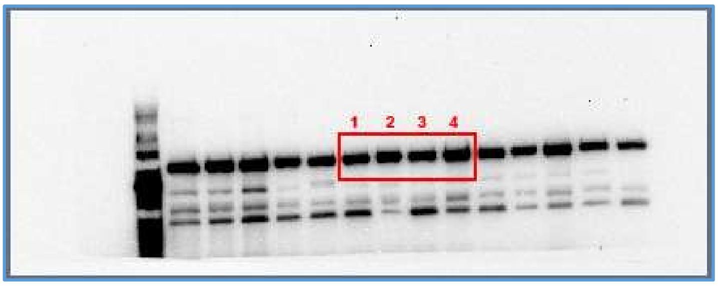

o

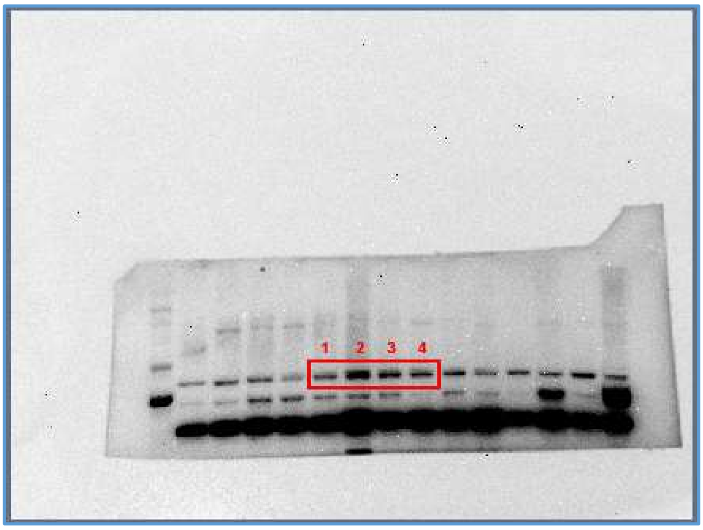

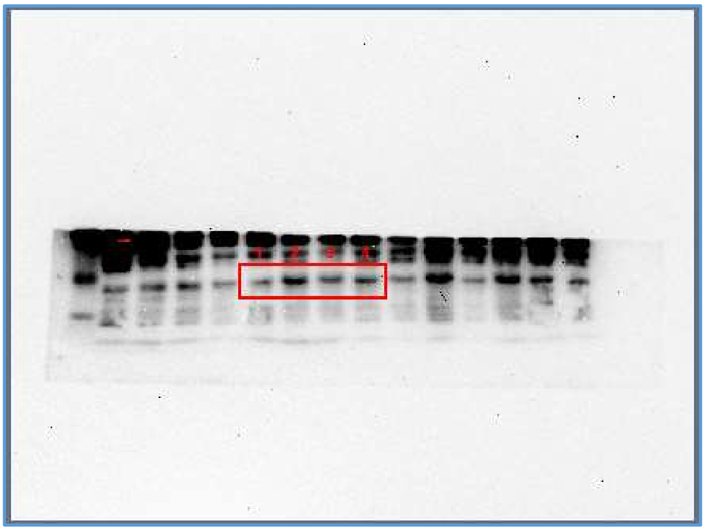

p

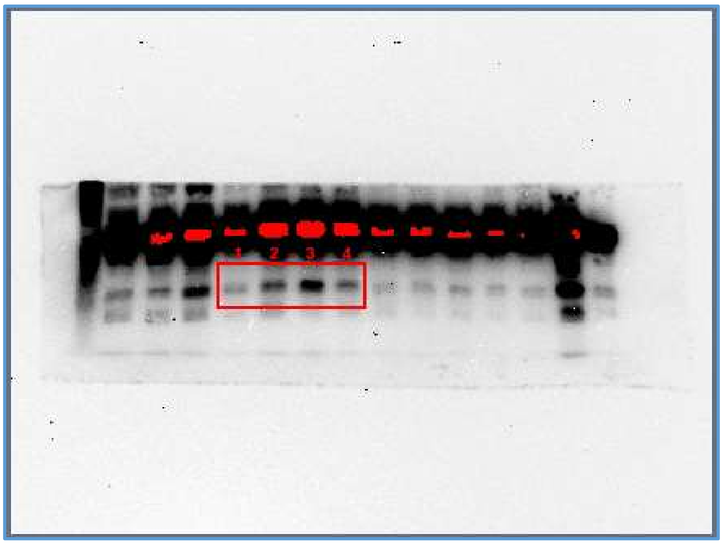

q

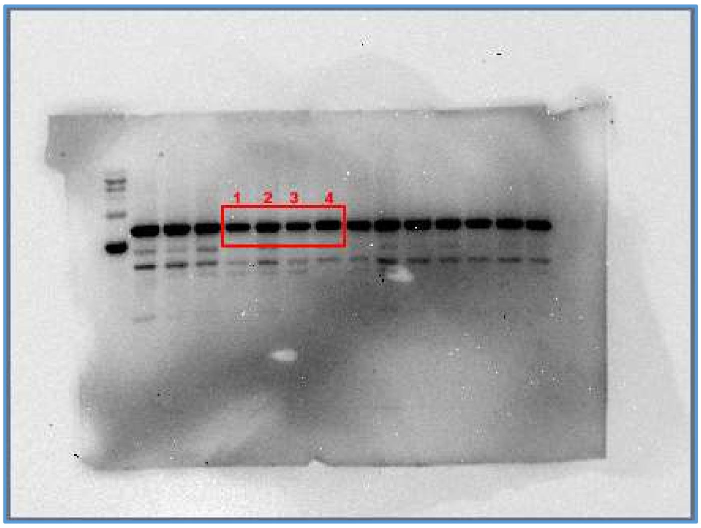

r
